# Generalized anxiety disorder and its associated factors among undergraduate health students of Chitwan District, Nepal

**DOI:** 10.1186/s12889-026-28645-x

**Published:** 2026-07-20

**Authors:** Susmita Thapa, Manish Rajbanshi, Subash Wagle, Bedana Sapkota, Richa Aryal, Lokendra Oli, Ashesh Malla Thakuri, Sanjeeb Shah

**Affiliations:** 1 Department of Public Health and Community Medicine, Chitwan Medical College, Tribhuvan University, Chitwan, Nepal; 2 Ministry of Health and Food Safety, Government of Nepal, Kathmandu, Nepal; 3 Central Department of Public Health, Institute of Medicine, Tribhuvan University, Kathmandu, Nepal; 4 Department of Public Health, Shree College of Technology, Purbanchal University, Chitwan, Nepal; 5 Department of Psychology, Padmakanya Multiple Campus, Tribhuvan University, Kathmandu, Nepal

**Keywords:** Generalized Anxiety Disorder, Undergraduate health students, Chitwan, Nepal

## Abstract

**Background:**

Generalized Anxiety Disorder (GAD), one of the most common anxiety disorders, is more prevalent among undergraduate health students than in the general population. Despite its adverse effects on academic performance and well-being, it often remains underrecognized in academic settings. Therefore, this study assessed GAD and its associated factors among undergraduate health students of Chitwan District, Nepal.

**Methodology:**

A cross-sectional study was conducted among 274 undergraduate health students of Chitwan Medical College using two-stage stratified random sampling. Data were collected through a self-administered questionnaire using the GAD-7 tool. Data were analyzed using IBM Statistical Package for Social Sciences (SPSS) Version 25. Chi-square tests and binary logistic regression were applied to identify associated factors. Variables with p-values < 0.2 were included into the multivariate logistic regression model. Model fit was assessed using the Hosmer-Lemeshow goodness of fit test and Nagelkerke R². All the tests were set at a 95% Confidence Interval (CI) and variables with a p-value of < 0.05 were considered statistically significant in this study.

**Results:**

The mean (± SD) age of participants was 21.2 ± 2.1 years. The prevalence of GAD was 52%. Among students with GAD, 64.8% had mild, 22.6% moderate, and 12.6% severe levels. The most commonly reported problems were academic difficulties (17.9%), bullying (10.6%), and physical abuse (8.1%). The study found significant associations between GAD and family type (AOR = 1.42, 95% CI: 1.09–2.14), perceived financial stress in the family (AOR = 2.20, 95% CI: 1.10–4.41), stressful life events (AOR = 2.40, 95% CI: 1.16–2.97), presence of psychosocial risk factors (AOR = 2.18, 95% CI: 1.09–3.37), smoking (AOR **=** 2.85, 95% CI: 1.03–4.89), and physical inactivity (AOR = 2.92, 95% CI: 1.25–6.84).

**Conclusion:**

The high prevalence of GAD among undergraduate health students highlights the important role of family, academic, financial, psychosocial, and lifestyle-related factors in students’ mental health. Strengthening family and psychosocial support, addressing financial stress, promoting healthy lifestyles, and helping students cope with study-related pressure and stressful life events may help reduce the burden of GAD. Universities should prioritize early screening, accessible mental health services, and stress management programs to improve students’ mental well-being.

**Supplementary Information:**

The online version contains supplementary material available at https://doi.org/10.1186/s12889-026-28645-x.

## Introduction

Generalized Anxiety Disorder (GAD) is defined in the Diagnostic and Statistical Manual of Mental Disorders, Fifth Edition (DSM-5) as an anxiety disorder characterized by excessive and difficult-to-control anxiety and worry that persists for at least six months [1]. Common symptoms include restlessness, difficulty concentrating, fatigue, muscle tension, irritability, and sleep disturbances [2]. The etiology of GAD is multifactorial, involving genetic, biological, environmental, and psychosocial factors, including trauma, family history, and neurotransmitter imbalance [2, 3]. Although the exact cause remains unclear, GAD can be effectively managed through psychotherapy, medication, and lifestyle modifications, such as regular physical activity, mindfulness, and adequate sleep [2, 4].

GAD is one of the most common anxiety disorders worldwide. Epidemiological studies have reported a lifetime prevalence of approximately 4–6% and a 12-month prevalence of around 2–3% in the general population [3, 5]. It is a significant public health concern because it adversely affects psychological well-being, daily functioning, academic performance, and quality of life [6]. University students enrolled in health-related disciplines have a higher prevalence of GAD than the general population, ranging from 17.5% in Australia [7], to 32.7% in South America [8], and up to 84.3% in Asian and African countries [9–13]. The demanding nature of health science education, including frequent examinations, clinical training, prolonged study hours, early professional responsibilities, and concerns about future career prospects, contributes to the higher risk of GAD among health students [8, 13, 14].

In Nepal, studies have reported anxiety prevalence among health students ranging from 22.7% to 53.9% [15–17]. Studies specifically assessing GAD have reported prevalence ranging from 47.7% to 67% among university and medical students [18, 19]. These variations may be attributed to differences in study design, timing of data collection, sample characteristics, and institutional support systems [17–19]. Despite the growing burden of mental health problems, many cases remain under-recognized and under-diagnosed because of limited mental health literacy, stigma, and poor help-seeking behavior [20, 21].

Although several studies in Nepal have examined anxiety among university students, most have focused on general anxiety symptoms or medical students, with limited attention to GAD across multiple undergraduate health disciplines. Undergraduate health students, including those in medicine, nursing, pharmacy, public health, and allied health sciences, experience similar academic and clinical stressors, such as demanding curricula, frequent examinations, clinical postings, and early professional responsibilities, which may place them at comparable risk of GAD. However, evidence regarding the prevalence of GAD and its associated factors across these disciplines in Nepal remains limited.

Addressing this gap is important for generating evidence to improve student mental health services and interventions. By assessing GAD and its associated academic, family, psychosocial, and lifestyle-related factors across undergraduate health disciplines, this study provides comprehensive, context-specific evidence to support the early identification of students at risk of GAD and inform targeted mental health interventions and institutional policies. Such evidence may contribute to Nepal’s efforts toward achieving Sustainable Development Goal (SDG) 3, particularly Target 3.4, by informing strategies to promote mental health and well-being among university students [22, 23]. Therefore, this study aimed to assess the prevalence of GAD and its associated factors among undergraduate health students in Chitwan District, Nepal.

## Materials and methodology

### Study design and setting

This study employed a cross-sectional design and was conducted in Chitwan District, Nepal. Chitwan is located in Bagmati Province and is an important educational and healthcare hub with several institutions offering undergraduate health science programs [24]. The district has two medical colleges: Chitwan Medical College (CMC) and College of Medical Sciences (COMS). CMC, established in 2006 and affiliated with Tribhuvan University, offers undergraduate and postgraduate programs in medicine and other health sciences and has more than 30,000 graduates [25].

Chitwan was selected as the study setting because it hosts multiple undergraduate health programs, providing access to a diverse population of undergraduate health students across different disciplines. Furthermore, no previous study has specifically assessed GAD among undergraduate health students in this district.

### Study population

The study population included undergraduate students of Chitwan Medical College enrolled in health-related disciplines: Bachelor of Medicine and Bachelor of Surgery (MBBS), Bachelor of Dental Surgery (BDS), Bachelor in Public Health (BPH), Bachelor of Nursing Science (BNS), Bachelor of Pharmacy (B. Pharm), Bachelor of Medical Laboratory Technology (BMLT) and Bachelor of Science in Nursing (B.Sc. Nursing).

These students were included because they are exposed to similar academic and clinical stressors and may share comparable mental health risk profiles. Students with a self-reported history of mental disorders, those who were pregnant or breastfeeding, and those unable to communicate during data collection were excluded.

### Sample size and sampling technique

The sample size was calculated using the single population proportion formula (n = Z^2^p q/d^2^) [26]. A prevalence estimate of 17.5% for GAD was obtained from a study among Australian university students [7]. Assuming a 95% confidence interval (CI), 5% margin of error, and 10% non-response rate, the minimum required sample size was 244.

The study population comprised all 1,087 undergraduate students enrolled at Chitwan Medical College (CMC), including MBBS (*n* = 372), BDS (*n* = 150), BSc Nursing (*n* = 148), B.Pharm (*n* = 122), BNS (*n* = 111), BPH (*n* = 97), and BMLT (*n* = 87), across four academic years. Student enrollment records were obtained from the administration office of CMC. The total number of students in each academic year was 274 (first year), 275 (second year), 274 (third year), and 264 (fourth year). Because data collection coincided with examinations, academic vacations, and student absenteeism, 310 students completed the questionnaire (first year = 68, second year = 84, third year = 78, fourth year = 80). To ensure equal representation across academic years, academic year was used as the sampling stratum. Since the first-year stratum had the lowest number of respondents (*n* = 68), 68 participants were randomly selected from each of the remaining academic-year strata using computer-generated random numbers based on student identification numbers. Two additional participants were then randomly selected from the remaining available respondents, resulting in a final sample of 274 students. (S1 Fig)

### Data collection and techniques

Data were collected between October 1 and November 15, 2023. A structured self-administered questionnaire was distributed in classroom settings after providing orientation and instructions. Participants completed the questionnaire independently, with clarification available from the research team when required. Prior to data collection, the questionnaire was pretested among 10% of the calculated sample size (*n* = 30) at the College of Medical Sciences (COMS), Chitwan. The pretest confirmed the clarity, internal consistency, reliability and comprehensibility of the questionnaire. Both English and Nepali versions of the questionnaire were used to ensure clarity and improve participant understanding. On average, participants required 30 min to complete the questionnaire. Prior to data collection, written permission was obtained from the Head of Department (HoD) of each faculty, and data collection was facilitated by faculty members and the research team.

### Tools and measures

The Generalized Anxiety Disorder (GAD-7) is a validated screening tool used to assess GAD and identify individuals who may require further clinical evaluation [27]. It is a standard instrument developed by Spitzer et al. [27]. The tool has a sensitivity of 89% and a specificity of 82% for assessing GAD. The scale consists of seven items rated on a 4-point Likert scale ranging from “not at all” to “nearly every day” [27] .

Each item is scored from 0 to 3, yielding a total score ranging from 0 to 21 [27]. For analysis, total scores were categorized as 0–4 (normal/no GAD), 5–9 (mild GAD), 10–14 (moderate GAD), and 15–21 (severe GAD) [27]. For binary logistic regression analysis, participants with a GAD-7 score of ≥ 5 were classified as having GAD, while those with a score < 5 were classified as not having GAD. This cutoff was selected in accordance with previous studies conducted among university students in Nepal and other Asian countries [10, 11, 13, 18, 19]. The questionnaire demonstrated good internal consistency during pretesting, with a Cronbach’s alpha of 0.89.

The study tool consisted of five sections: socio-demographic characteristics, family-related factors, academic-related factors, lifestyle-related factors, and GAD-related items. Socio-demographic variables included age, sex, ethnicity, marital status, department, family type, living condition, family size, and parental education. Family-related variables included parental relationship status, family history of psychiatric disorders, reported financial stress, stressful life events, chronic disease, parenting style, and parent-child relationship.

Academic-related factors included career choice satisfaction, academic performance satisfaction, primary companion, perceived study-related pressure, perceived coping strategy, and psychosocial risk factors. Psychosocial risk factors included exam anxiety, loneliness, difficulty coping with studies, pressure to succeed, time management issues, work-life-study imbalance, lack of confidence, body image concerns, relationship issues, and homesickness. Each item was recorded as a yes/no response, and a composite score was generated. Participants scoring more than 5 were categorized as having psychosocial risk factors. This was an exploratory, non-validated index used for screening purposes.

Lifestyle-related factors included extracurricular activities involvement, duration of smart device use, smoking, alcohol consumption, tea/coffee consumption, leisure activities, and physical activity. The GAD-7 items assessed symptoms such as feeling nervous, anxious, or on edge; inability to control worrying; excessive worry about different things; difficulty relaxing; restlessness; irritability; and feeling afraid as if something awful might happen.

### Data management and analysis

Collected data were entered, compiled, cleaned, and cross-checked using EpiData version 3.1 and then exported to IBM Statistical Package for Social Sciences (SPSS) Version 25 for analysis. Descriptive statistics, including numbers , percentages, mean, and standard deviation, were used to summarize the data. Chi-square tests and bivariate binary logistic regression were performed for bivariate analysis. Variables with a p-value < 0.2 were entered into the multivariable logistic regression model. Multicollinearity among the independent variables was assessed using the Variance Inflation Factor (VIF), and all variables had VIF values < 5, indicating no significant multicollinearity. Multivariate logistic regression was used to identify factors associated with GAD. Model fit was assessed using the Hosmer-Lemeshow goodness-of-fit test (*p* = 0.615), indicating an adequate model fit. The Nagelkerke R² value was 0.256. The model demonstrated acceptable discriminatory ability, with an Area Under the Receiver Operating Characteristic (ROC) curve (AUC) of 0.764. Adjusted odds ratios (AOR) with 95% confidence intervals (CI) were reported, and a p-value < 0.05 was considered statistically significant.

### Ethical consideration

This study was reviewed and approved by the Ethical Review Committee of Chitwan Medical College [Reference no: 080/081–097]. Written permission was obtained from the administration of CMC. All procedures involving human participants were conducted in accordance with the ethical principles of the Declaration of Helsinki and the Institutional Review Committee (IRC) of Chitwan Medical College guidelines and regulations. Written informed consent was obtained from all participants after explaining the purpose of the study. Confidentiality and anonymity were strictly maintained throughout the study. Participants were informed of their right to refuse or withdraw from the study at any stage without any consequences.

## Results

### Socio-demographic characteristics of the participants

In the current study, a total of 274 undergraduate health students participated. The mean (± SD) age of the participants was 21.2 ± 2.1 years (range: 18–30 years). Most participants were female (65.3%), Brahmin (39.8%), and unmarried (95.6%). Approximately one-third (32.8%) were enrolled in the MBBS program, and 39.1% resided in hostels. The majority belonged to nuclear families (73.0%) with more than four family members (61.7%). Regarding parental education, 42.3% of fathers had attained a bachelor’s degree or higher, whereas most mothers had completed secondary-level education (39.1%) (Table 1).

**Table 1:** Socio-demographic characteristics of the participants (*n* = 274)

Characteristics	Number (*n*)	Percentage (%)
Age (Mean ± SD= 21.2 ± 2.1, Range = 18–30)		
≤ 19	34	12.4
> 19	240	87.6
Sex		
Female	179	65.3
Male	95	34.7
Ethnicity		
Brahmin	109	39.8
Chhetri	60	21.9
Madhesi	59	21.5
Janajati	43	15.7
Others (Dalit/Muslim)	3	1.1
Marital status		
Unmarried	262	95.6
Married	12	4.4
Department		
MBBS	90	32.8
BDS	38	13.9
BSC. Nursing	35	12.8
B. Pharm	31	11.3
BNS	28	10.2
BPH	27	9.9
BMLT	25	9.1
Living condition		
Hostel	107	39.1
Family	94	34.3
Alone	33	12.0
Friends	27	9.9
Others (Relatives, Husband/wife)	13	4.7
Family type		
Nuclear	200	73.0
Joint/Extended	74	27.0
Family size		
≤ 4	105	38.3
> 4	169	61.7
Educational status of father		
Illiterate	3	1.1
Can read and write	14	5.1
Basic primary level	43	15.4
Secondary level	98	36.1
Bachelor and above	116	42.3
Educational status of mother		
Illiterate	30	10.9
Can read and write	21	7.7
Basic primary level	49	17.8
Secondary level	107	39.1
Bachelor and above	67	24.5

### Family-related characteristics of the participants

Most participants reported that their parents were living together (96.0%). A family history of psychiatric disorders was absent in 94.9% of participants, and 61.3% perceived financial stress within their family. Nearly half reported a mixed parenting approach (both strict and permissive) (48.9%), while 71.9% described their relationship with their parents as very good. Furthermore, 75.2% had not experienced stressful life events, and 96.4% reported no chronic disease in the family (Table 2).

**Table 2:** Family-related characteristics of the participants (*n* = 274)

Characteristics	Number (*n*)	Percentage (%)
Relationship status of parents		
Together	263	96.0
Not together	11	4.0
Family history of any psychiatric disorder		
Yes	14	5.1
No	260	94.9
Financial stress in family		
Yes	168	61.3
No	106	38.7
Parenting approach		
Strict	36	13.1
Permissive	99	36.2
Both strict and permissive	134	48.9
Neglectful	5	1.8
Parent-child relationship		
Poor	5	1.8
Fair	9	3.3
Good	63	23.0
Very good	197	71.9
Stressful life events in the family		
Yes	68	24.8
No	206	75.2
Presence of chronic disease		
Yes	10	3.6
No	264	96.4

### Academic-related characteristics of the participants

The majority of participants were satisfied with their career choice (89.1%), whereas just over half were satisfied with their academic performance (51.8%). Most were non-scholarship students (88.3%). Friends (37.6%) and family members (36.9%) were the most commonly reported primary companions. Moderate study-related pressure was reported by 58.8% of participants. Nearly half had good coping strategies (47.1%), and 59.9% reported the presence of psychosocial risk factors (Table 3).

**Table 3:** Academic-related characteristics of the participants (*n* = 274)

Characteristics	Number (*n*)	Percentage (%)
Career choice satisfaction		
Yes	244	89.1
No	30	10.9
Academic performance satisfaction		
Yes	142	51.8
No	132	48.2
Type of academic funding		
Full scholarship	32	11.7
Non-scholarship	242	88.3
Primary companion		
Family	101	36.9
Friends	103	37.6
Alone	70	25.5
Perceived pressure related to the study		
Not at all	28	10.2
Low	19	6.9
Moderate	161	58.8
High	66	24.1
Coping strategy		
Poor	29	10.6
Fair	79	28.8
Good	129	47.1
Excellent	37	13.5
Presence of psychosocial risk factors		
Yes	164	59.9
No	110	40.1

### Problems faced by students during their lifetime

Among the problems faced by participants, difficulty in school/college was the most commonly reported (17.9%), followed by bullying (10.6%), physical abuse (8.1%), sexual harassment (8.1%), and early separation from parents (5.9%). (S2 Fig)

### Lifestyle-related characteristics

Most participants reported occasional involvement in extracurricular activities (60.2%) and daily smart device use of two hours or more (93.4%). Smoking and alcohol consumption were reported by 12.8% and 31.0% of participants, respectively. Coffee and tea were consumed by 72.6% and 78.1% of participants, respectively. Nearly half engaged in leisure activities occasionally (47.4%), while 74.1% reported participating in physical activity (Table 4).

**Table 4:** Lifestyle-related characteristics of the participants (*n* = 274)

Characteristics	Number (*n*)	Percentage (%)
Extracurricular activities involvement		
Occasionally	165	60.2
Often	46	16.8
Regularly	37	13.5
Never	26	9.5
Duration of smart device use		
< 2 h	18	6.6
≥ 2 h	256	93.4
Smoking		
Yes	35	12.8
No	239	87.2
Frequency of smoking, *n* = 35		
Occasional	22	62.9
Often	8	22.9
Regular	5	14.2
Alcohol consumption		
Yes	85	31.0
No	189	69.0
Frequency of alcohol consumption, *n* = 85		
Occasional	74	87.1
Often	6	7.1
Regular	5	5.8
Coffee consumption		
Yes	199	72.6
No	75	27.4
Frequency of coffee consumption, *n* = 199		
Occasional	88	44.2
Often	85	42.7
Regular	24	13.1
Tea consumption		
Yes	214	78.1
No	60	21.9
Frequency of tea consumption, *n* = 214		
Occasional	52	24.3
Often	69	32.2
Regular	93	43.5
Frequency of leisure activities		
Occasionally	130	47.4
Often	89	32.6
Regularly	33	12.0
Never	22	8.0
Physical activity		
Yes	203	74.1
No	71	25.9

### GAD-related symptoms experienced by respondents

The most frequently reported GAD-7 symptoms were restlessness (66.7%) and feeling nervous or on edge (51.4%), with most participants experiencing these symptoms on several days. A smaller proportion reported symptoms nearly every day, such as feeling something awful might happen (13.5%), trouble relaxing (14.2%), and irritability (9.9%). (S3 Fig)

### GAD among undergraduate health students

Overall, 52.0% of participants had GAD. Among those with GAD, 64.8% had mild, 22.6% had moderate, and 12.6% had severe levels of GAD. (S4 Fig)

### Factors associated with socio-demographic characteristics of the participants

Multivariable logistic regression showed that family type was significantly associated with GAD. Participants from nuclear families had significantly higher odds of having GAD than those from joint or extended families (AOR = 1.42, 95% CI: 1.09–2.14) (Table 5).

**Table 5:** Factors associated with socio-demographic characteristics of the participants (*n* = 274)

Characteristics	Prevalence of GAD				Multivariate logistic regression	
	Yes	No	COR, 95% CI	*p*-value	AOR, 95% CI	*p*-value
	*n* (%)	*n* (%)				
Age						
≤ 19	21 (61.8)	13 (38.2)	1.37 (0.65–2.91)			
> 19	138 (57.5)	102 (42.5)	Ref	0.401		
Sex						
Female	115 (63.9)	65 (36.1)	2.01 (1.21–3.33)		1.54 (0.83–2.87)	
Male	44 (46.8)	50 (53.2)	Ref	0.007	Ref	0.164
Ethnicity						
Madhesi	29 (48.3)	31 (51.7)	0.80 (0.35–1.84)		0.92 (0.37-2.18)	
Others (Janajati/Dalit/ Muslim)	38 (65.5)	20 (34.5)	0.65 (0.25–1.66)		0.58 (0.21-1.57)	
Brahmin/Chhetri	92 (59.0)	64 (41.0)	Ref	0.160	Ref	0.646
Marital Status						
Unmarried	152 (58.0)	110 (42.0)	1.01 (0.31–3.27)			
Married	7 (58.3)	5 (41.7)	Ref	0.983		
Department						
BSC. Nursing/BNS	37 (58.7)	26 (41.3)	0.99 (0.53–1.83)			
Others	47 (56.6)	36 (43.4)	1.08 (0.62–1.89)			
MBBS/BDS	75 (58.6)	53 (41.4)	Ref	0.953		
Living condition						
Alone	21 (63.6)	12 (36.4)	0.67 (0.29–1.53)			
Hostel	61 (57.0)	46 (43.0)	0.89 (0.51–1.56)			
Others	26 (65.0)	14 (35.0)	0.63 (0.29–1.37)			
Family	51 (54.3)	43 (45.7)	Ref	0.613		
Family type						
Nuclear	125 (62.5)	75 (37.5)	1.96 (1.14–3.36)		1.42 (1.09–2.14)	
Joint/Extended	34 (45.9)	40 (54.1)	Ref	0.014	Ref	0.007
Family size						
> 4	97 (57.4)	72 (42.6)	0.93 (0.57–1.53)			
≤ 4	62 (59.0)	43 (41.0)	Ref	0.788		

*Ref*: Reference category, *COR *Crude Odds Ratio, *AOR *Adjusted Odds Ratio

*p-value statistically significant (<0.05)

### Factors associated with family-related characteristics of the participants

Perceived financial stress in the family and the presence of stressful life events were significantly associated with GAD. Participants who perceived financial stress in their family had more than twice the odds of having GAD compared to those who did not (AOR = 2.20, 95% CI: 1.10–4.41). Similarly, participants who reported stressful life events in the family had higher odds of GAD than those who did not (AOR = 2.40, 95% CI: 1.16–2.97) (Table 6).

**Table 6:** Factors associated with family-related characteristics of the participants (*n* = 274)

Characteristics	Prevalence of GAD				Multivariate logistic regression	
	Yes	No	COR, 95% CI	*p*-value	AOR, 95% CI	*p*-value
	*n* (%)	*n* (%)				
Relationship status of parents						
Together	151 (57.4)	112 (42.6)	0.50 (0.13–1.94)			
Not together	8 (72.7)	3 (27.3)	Ref	0.322		
Family history of any psychiatric disorder						
Yes	12 (85.7)	2 (14.3)	4.61 (1.01–21.02)		2.41 (0.5–10.8)	
No	147 (56.5)	113 (43.5)	Ref	0.048	Ref	0.405
Perceived financial stress in family						
Yes	84 (50.0)	84 (50.0)	2.41 (1.24–5.69)		2.20 (1.10–4.41)	
No	31 (29.2)	75 (70.8)	Ref	0.001	Ref	0.026
Parenting approach						
Both strict and permissive	81 (60.4)	53 (39.6)	0.64 (0.38–1.08)		0.90 (0.44–1.85)	
Strict	27 (75.0)	9 (25.0)	0.32 (0.13–0.76)		0.42 (0.12–1.38)	
Neglectful	2 (40.0)	3 (60.0)	1.47 (0.23–9.18)		3.49 (0.19–63.93)	
Permissive	49 (49.5)	50 (50.5)	Ref	0.048	Ref	0.414
Parent-child relationship						
Fair	11 (78.6)	3 (21.4)	0.28 (0.07–1.06)		0.42 (0.04–4.38)	
Good	47 (74.6)	16 (25.4)	0.35 (0.19–0.67)		0.41 (0.28–1.58)	
Very good	101 (51.3)	96 (48.7)	Ref	0.002	Ref	0.286
Stressful life events in family						
Yes	54 (79.4)	14 (20.6)	3.71 (1.94–7.09)		2.40 (1.16–2.97)	
No	105 (51)	101 (49)	Ref	< 0.001	Ref	0.043
Presence of chronic disease						
Yes	6 (60.0)	4 (40.0)	1.08 (0.30–3.94)			
No	153 (58)	111 (42)	Ref	0.898		

*Ref*: Reference category, *COR *Crude Odds Ratio, *AOR *Adjusted Odds Ratio

*p-value statistically significant (<0.05)

### Factors associated with academic-related characteristics of the participants

Perceived study-related pressure and psychosocial risk factors were significantly associated with GAD. Participants reporting moderate study-related pressure had significantly higher odds of GAD than those reporting no study-related pressure (AOR = 4.60, 95% CI: 1.82–11.60). Similarly, participants with psychosocial risk factors had more than twice the odds of GAD compared with those without psychosocial risk factors (AOR = 2.18, 95% CI: 1.09–3.37) (Table 7).

**Table 7:** Factors associated with academic-related characteristics of the participants (*n* = 274)

Characteristics	Prevalence of GAD				Multivariate logistic regression	
	Yes	No	COR, 95% CI	*p*-value	AOR, 95% CI	*p*-value
	*n* (%)	*n* (%)				
Career choice satisfaction						
Yes	136 (55.7)	108 (44.3)	0.38 (0.15–0.92)		0.73 (0.22–2.40)	
No	23 (76.7)	7 (23.3)	Ref	0.028	Ref	0.606
Academic performance satisfaction						
Yes	65 (45.8)	77 (54.2)	0.34 (0.20–0.56)		1.43 (0.69–2.95)	
No	94 (71.2)	38 (28.8)	Ref	< 0.001	Ref	0.326
Type of academic funding						
Non-scholarship	139 (57.4)	103 (42.6)	0.81 (0.37–1.73)			
Full scholarship	20 (62.5)	12 (37.5)	Ref	0.586		
Primary companion						
Alone	56 (80)	14 (20)	0.38 (0.19–0.73)		2.45 (0.72–3.96.)	
Friends	53 (51.5)	50 (48.5)	0.82 (0.47–1.42)		1.07 (0.43–2.62)	
Family	50 (49.5)	51 (50.5)	Ref	0.013	Ref	0.681
Perceived pressure related to study						
High	53 (80.3)	13 (19.7)	4.70 (1.83–12.27)		2.25 (0.55–9.21)	
Low	8 (42.1)	11 (57.9)	5.60 (1.87–16.74)		7.31 (1.66–32.12)	
Moderate	85 (52.8)	76 (47.2)	3.64 (1.84–7.20)		4.60 (1.82–11.60)	
Not at all	13 (46.4)	15 (53.6)	Ref	0.001	Ref	0.006
Coping strategy						
Excellent	17 (45.9)	20 (54.1)	3.69 (1.27–10.76)		2.56 (0.15–5.29)	
Good	66 (51.2)	63 (48.8)	3.00 (1.19–7.51)		0.81 (0.21–3.03)	
Fair	54 (68.4)	25 (31.6)	1.45 (0.55–3.85)		0.57 (0.14–2.21)	
Poor	22 (75.9)	7 (24.1)	Ref	0.009	Ref	0.754
Presence of psychosocial risk factors						
Yes	126 (76.8)	38 (23.2)	7.73 (4.48–13.35)		2.18 (1.09–3.37)	
No	33 (30.0)	77 (70.0)	Ref	< 0.001	Ref	< 0.001

*Ref*: Reference category, *COR *Crude Odds Ratio, *AOR *Adjusted Odds Ratio

*p-value statistically significant (<0.05)

### Factors associated with lifestyle-related characteristics of the participants

Smoking and physical inactivity were significantly associated with GAD. Smokers had higher odds of GAD than non-smokers (AOR = 2.85, 95% CI: 1.03–4.89). Likewise, participants who did not engage in physical activity had nearly three times higher odds of GAD than those who were physically active (AOR = 2.92, 95% CI: 1.25–6.84) (Table 8).

**Table 8:** Factors associated with lifestyle-related characteristics of the participants (*n* = 274)

Characteristics	Prevalence of GAD				Multivariate logistic regression	
	Yes	No	COR, 95% CI	*p*-value	AOR, 95% CI	*p*-value
	*n* (%)	*n* (%)				
Extracurricular activities involvement						
Regularly	16 (43.2)	21 (56.8)	2.95 (1.02–8.49)		1.25 (0.23–6.56)	
Often	21 (45.7)	25 (54.3)	2.67 (0.97–7.39)		1.65 (0.20–4.57)	
Occasionally	104 (63)	61 (37)	1.32 (0.54–3.21)		0.61 (0.15–2.49)	
Never	18 (69.2)	8 (30.8)	Ref	0.029	Ref	0.483
Duration of smart devices use						
< 2 h	8 (44.4)	10 (55.6)	0.55 (0.21–1.45)		1.08 (0.27–4.24)	
≥ 2 h	151 (59)	105 (41)	Ref	0.232	Ref	0.910
Smoking						
Yes	24 (68.6)	11 (31.4)	1.68 (0.78–3.58)		2.85 (1.03–4.89)	
No	135 (56.5)	104 (43.5)	Ref	0.176	Ref	0.039
Alcohol consumption						
Yes	53 (62.4)	32 (37.6)	1.29 (0.76–2.19)			
No	106 (56.1)	83 (43.9)	Ref	0.331		
Coffee consumption						
Yes	111 (55.8)	88 (44.2)	0.71 (0.41–1.22)		1.27 (0.59–2.72)	
No	48 (64)	27 (36)	Ref	0.219	Ref	0.530
Tea consumption						
Yes	126 (58.9)	88 (41.1)	1.17 (0.65–2.08)			
No	33 (55)	27 (45)	Ref	0.591		
Frequency of leisure activities						
Regularly	14 (42.4)	19 (57.6)	2.90 (0.93–9.02)		1.36 (0.23–8.07)	
Often	46 (51.7)	43 (48.3)	2.00 (0.74–5.38)		0.76 (0.15–3.83)	
Occasionally	84 (64.6)	46 (35.4)	1.17 (0.44–3.08)		0.85 (0.18–4.05)	
Never	15 (68.2)	7 (31.8)	Ref	0.049	Ref	0.760
Physical activity						
No	51 (71.8)	20 (28.2)	2.24 (1.15–4.72)		2.92 (1.25–6.84)	
Yes	108 (53.2)	95 (46.8)	Ref	0.007	Ref	0.013

*Ref*: Reference category, *COR *Crude Odds Ratio, *AOR *Adjusted Odds Ratio

*p-value statistically significant (<0.05)

## Discussion

The burden of anxiety disorders has increased in Nepal over recent decades, while GAD remains underrecognized and undertreated [5, 28]. GAD can impair attention, concentration, working memory, and perceptual-motor function, which may adversely affect the academic performance and future clinical practice of health science students [3].

This study demonstrated that 52% of participants experienced GAD, with most reporting mild levels of GAD. This finding is consistent with studies conducted among Iranian nurses (55.1%) [29] and university students in Brazil (52%) [8], where mild GAD were also predominant. However, the prevalence observed in this study was lower than that reported among students in a Saudi university (84.3%) [11], Majmaah University (67.5%) [12] and Nepal (67%) [19]. These findings indicate that GAD represents a substantial mental health burden among undergraduate health students. The high prevalence observed in this study may reflect the demanding academic and clinical environment of health science education, which can contribute to persistent stress and excessive worry, key features of GAD [3, 30]. In Nepal, barriers to mental health care, including stigma surrounding help-seeking and the limited availability of mental health services, may further contribute to this burden [21, 28, 31]. These findings underscore the importance of early identification and timely mental health support for undergraduate health students [32].

Furthermore, findings from studies involving Australian (17.5%) [7] and US students (30.6%) [33] contrast with those of this study, revealing lower prevalence rates. The lower prevalence in developed countries may be explained by better access to mental health resources such as counseling services, early intervention programs, and digital support tools [31]; higher mental health literacy and reduced stigma encouraging help-seeking behavior [32]; stronger economies that reduce financial stress among students [23]; and the availability of university-based mental health promotion and early intervention programs [32]. Overall, variations in prevalence across studies may also reflect differences in participant characteristics, academic settings, periods of data collection, sampling strategies, and assessment instruments (i.e. GAD-2 and GAD-7) [7, 8, 11, 12, 19].

Students who belonged to nuclear families reported significantly higher odds of GAD than those from joint/extended families. One possible explanation is that students from nuclear families may have fewer readily available sources of emotional and practical support during stressful situations, which may limit their ability to cope effectively with stress [34]. Comparable findings have been observed among university students in Bangladesh, where students from nuclear families had more severe anxiety symptoms than those from joint families [35]. In contrast, students from joint/extended families may benefit from greater perceived family support, which can enhance coping with stress and reduce vulnerability to anxiety [36].

Students who experienced financial stress in their families had 2.2 times higher odds of developing GAD compared to those who did not. This association aligns with studies conducted among medical students in Australia [7] and Majmaah [12]. Financial stress increases psychological distress by creating concerns about tuition fees, living expenses, and academic performance, which can lead to feelings of inadequacy and fear of failure [37]. It also negatively affects social relationships, as students may compare themselves with financially better-off peers, leading to feelings of shame, frustration, and social isolation [38]. Persistent financial insecurity may also increase uncertainty regarding educational continuity and future career opportunities [38], thereby sustaining excessive and persistent worry, a hallmark feature of GAD [3].

Students who faced stressful life events in their family had 2.4 times higher odds of GAD than those who did not. This association is consistent with previous study demonstrating that exposure to stressful life events increases the risk of generalized anxiety disorder [39]. This may be because exposure to stressful or adverse life events increases psychological vulnerability, leading to heightened emotional distress and difficulty coping with academic and personal demands [39]. Such experiences can overwhelm an individual’s coping capacity and contribute to the development of anxiety symptoms [40].

Students who perceived study-related pressure had higher odds of GAD than those who did not. A comparable association has been reported among Australian university students [7]. Constant academic demands may overwhelm students, leading to chronic worry, fear of failure, and difficulties in managing stress [41]. In addition, students experiencing greater academic pressure may adopt maladaptive coping strategies, such as procrastination or avoidance, which may further exacerbate anxiety symptoms [42].

This study found that psychosocial risk factors were significantly associated with GAD among undergraduate health students. This is consistent with findings among Australian university students, where psychosocial stressors, including pressure to succeed and difficulty coping with study, were significantly associated with GAD [7]. Students reporting factors such as exam anxiety, loneliness, difficulty coping with studies, and pressure to succeed were more likely to experience GAD. This association may be explained by the cumulative effect of these stressors on psychological well-being, which can increase emotional distress, impair adaptive coping, and contribute to persistent worry, a core feature of GAD [3, 42]. Furthermore, inadequate social support and ineffective coping strategies may reduce resilience to academic and personal stressors, thereby increasing vulnerability to anxiety symptoms [36, 42].

Smoking was significantly associated with GAD, with smokers having 2.85 times higher odds compared to non-smokers. This may be explained by the short-term anxiolytic effects of nicotine, followed by rebound anxiety and withdrawal symptoms that worsen overall anxiety levels over time [43]. Students may adopt smoking as a coping mechanism for academic and clinical stress; however, this maladaptive behavior may ultimately increase psychological distress [43]. A similar finding was observed among medical students in Saudi [11] and Tunisia [44].

Students who were not involved in physical activity had nearly three times higher odds of experiencing GAD than those who were physically active. Similar findings have been reported among students, where lower levels of physical activity were associated with higher anxiety [45]. Regular physical activity reduces anxiety by promoting the release of endorphins and regulating stress hormones such as cortisol [46]. It also enhances self-esteem, social connectedness, and emotional well-being, thereby reducing anxiety [46, 47].

The findings highlight context-specific factors associated with GAD among undergraduate health students, particularly modifiable factors such as study-related pressure and physical inactivity. These findings may help universities identify students at risk and inform targeted mental health promotion and early intervention strategies.

## Conclusion

This study found a high prevalence of generalized anxiety disorder among undergraduate health students. Family type, financial stress, stressful life events, study-related pressure, psychosocial risk factors, smoking, and physical inactivity were significantly associated with GAD, highlighting the multifactorial nature of GAD. These findings highlight the need for targeted, student-centered interventions, including early screening for anxiety, accessible mental health services, stress management and coping skills training, promotion of physical activity, and measures to address psychosocial challenges. In addition, institutional and policy-level efforts to reduce financial stress and foster supportive learning environments may contribute to improving students’ mental well-being and academic success.

### Strengths and limitations

This study utilized a valid and widely used screening tool (GAD-7), ensuring reliable assessment of GAD. It also assessed a broad range of academic, financial, family, behavioral, and psychosocial factors, providing a comprehensive understanding of GAD among undergraduate health students. Despite these strengths, several limitations should be considered. First, the cross-sectional design precludes establishing causal relationships between the identified factors and GAD. Second, the use of self-reported data may have introduced reporting and social desirability bias. Data collection in a classroom setting may also have influenced participants’ responses. Furthermore, as the study was conducted in a single medical college in Chitwan, Nepal, the findings may not be generalizable to students from other institutions or regions. Finally, as comparable Nepalese data were unavailable at the time of sample size calculation, the prevalence estimate was based on an Australian study, which may limit its applicability to the Nepalese context.

## Supplementary Information

Supplementary Material 1.

Supplementary Material 2.

Supplementary Material 3.

Supplementary Material 4.

## Data Availability

The datasets generated and/or analyzed during the current study are not publicly available due to ethical restrictions and participant confidentiality but are available from the corresponding author on reasonable request. Requests for data access may also be subject to approval by the relevant institutional ethics committee.

## Abbreviations

AOR: Adjusted Odds Ratio
BDS: Bachelor of Dental Surgery
BMLT: Bachelor of Medical Laboratory Technology
BNS: Bachelor of Nursing Science
BPH: Bachelor in Public Health
B.Pharm: Bachelor of Pharmacy
B.Sc. Nursing: Bachelor of Science in Nursing
CI: Confidence Interval
CMC: Chitwan Medical College
COMS: College of Medical Sciences
COR: Crude Odds Ratio
GAD: Generalized Anxiety Disorder
HoD: Head of Department
IRC: Institutional Review Committee
MBBS: Bachelor of Medicine and Bachelor of Surgery
p-value: Probability value
SD: Standard Deviation
SPSS: Statistical Package for the Social Sciences
